# Enhancing amplification of late‐outgrowth endothelial cells by bilobalide

**DOI:** 10.1111/jcmm.13609

**Published:** 2018-03-22

**Authors:** Shuqin Liu, Xiaoye Hou, Lina Chen, Hao Hu, Qiang Sun, Feng Zhao, Chuanhao Liu

**Affiliations:** ^1^ Department of Pharmacology School of Basic Medical Sciences Xi'an Jiaotong University Health Science Center Key Laboratory of Environment and Genes Related to Diseases (Xi'an Jiaotong University) Ministry of Education of China Xi'an Shaanxi China; ^2^ The Basic Medical Central Laboratory School of Basic Medical Sciences Xi'an Jiaotong University Health Science Center Xi'an Shaanxi China

**Keywords:** bilobalide, endothelial nitric oxide synthase, late‐outgrowth endothelial cells, migration, nitric oxide, proliferation

## Abstract

Transfusion of autologous late‐outgrowth endothelial cells (OECs) is a promising treatment for restenosis after revascularization. Preparing cells by in vitro amplification is a key step to implement the therapy. This study aimed to demonstrate that bilobalide, a terpenoid, enhances the OEC amplification. Human‐, rabbit‐ and rat OECs and a mouse femoral artery injury model were used. Expanding OECs used endothelial growth medium‐2 as the standard culture medium while exploring the mechanisms used endothelial basal medium‐2. Proliferation assay used MTT method and BrdU method. Migration assay used the modified Boyden chamber. Intracellular nitric oxide, superoxide anion, hydroxyl radical/peroxynitrite and H_2_O_2_ were quantified with DAF‐FM DA, dihydroethidium, hydroxyphenyl fluorescein and a H_2_O_2_ assay kit, respectively. Activated ERK1/2 and eNOS were tested with the Western blot. Bilobalide concentration‐dependently enhanced OEC number increase in vitro. Transfusion of bilobalide‐based human OECs into femoral injured athymia nude mouse reduced the intimal hyperplasia. Bilobalide promoted OEC proliferation and migration and increased the intracellular nitric oxide level. L‐NAME, a NOS inhibitor, inhibits but not abolishes OEC proliferation, migration and ERK1/2 activation. Bilobalide concentration‐dependently enhanced the eNOS Ser‐1177 phosphorylation and Thr‐495 dephosphorylation in activated OECs. Bilobalide alleviates the increase in hydroxyl radical/peroxynitrite, superoxide anion and H_2_O_2_ in proliferating OECs. In conclusion, nitric oxide plays a partial role in OEC proliferation and migration; bilobalide increases OEC nitric oxide production and decreases nitric oxide depletion, promoting the OEC number increase; Bilobalide‐based OECs are active in vivo. The findings may simplify the preparation of OECs, facilitating the implementation of the autologous‐OECs‐transfusion therapy.

## 1 INTRODUCTION

Late‐outgrowth endothelial cells (OECs) also known as bone marrow (BM)‐derived ECs are a subtype of endothelial progenitor cells. OECs exist in many organs but are rare anywhere.^1^, ^3^ It is difficult to selectively mobilize sufficient OECs to the circulating blood and the diseased organs. We and other research groups have demonstrated that OECs directly participate in the re‐endothelialization and by a paracrine pathway inhibit the migration and proliferation of vascular smooth muscle cells; transfusion of sufficient OECs soon after vascular injury may reduce neointima formation,^4^, ^6^ opening a new way to inhibit the restenosis process after revascularization procedures such as percutaneous balloon angioplasty, stent implantation or atherectomy. In the forementioned three studies, we transfused autologous OECs that were prepared by in vitro amplification to the experimental rabbits while the two other research groups delivered allogeneic OECs to the immunodeficient mice. Allogeneic OECs fails to prevent intimal hyperplasia in immunity‐intact rats.^7^

Expanding OECs is a key step to implement the autologous‐OECs‐therapy. However, as we know, all the current expansion methods 1, 8 have low efficiencies, and getting seed OECs demands a large amount of peripheral blood (PB) or BM. Hence, it is useful to enhance the OEC expansion. As OECs have endothelial nitric oxide synthase (eNOS) and can proliferate whereas the early‐outgrowth endothelial progenitor cells lack eNOS and do not proliferate,^9^, ^10^ we hypothesized that nitric oxide is involved in OEC proliferation, and additives that can modulate the eNOS/nitric oxide pathway may accelerate the OEC amplification.

Bilobalide, a sesquiterpenoid having a 15‐carbon skeleton (Figure S1), is originally found in *Ginkgo biloba* leaves. *Ginkgo biloba* leaf is widely used in traditional Chinese medicine, and its standardized extract EGb 761 has been used worldwide because of multifaceted pharmacological benefits.^11^ Bilobalide accounts for 2.6%‐3.2% of EGb 761 and contributes to EGb 761's vasodilating effect.^12^ Although there is no report of using bilobalide alone in clinic, animal experiments have attested that bilobalide increases cerebral blood flow 13 and decreases arterial blood pressure, especially the diastolic pressure.^14^, ^15^ An in vitro study using aorta ring strips reveals that the vasorelaxation of bilobalide could be reduced significantly by NOS inhibitors.^16^ Bilobalide decreases the activity or expression of iNOS in some non‐vascular cells or tissues.^17^, ^19^ Our second hypothesis was that bilobalide modulates eNOS/nitric oxide pathway, promoting the OEC proliferation.

This article reports our verification of the hypotheses. We find that nitric oxide plays a partial role in OEC proliferation and migration; bilobalide promotes eNOS activation and superoxide scavenging to increase intracellular nitric oxide, enhancing the OEC expansion; bilobalide‐based OECs are active in vivo.

## 2 MATERIALS AND METHODS

### 2.1 Ethics

The procedures of the study received ethics approval from the Ethical Committee for Medical and Biological Research at Xi'an Jiaotong University (No. 2012‐0061) on March 10, 2012. Five human blood donators are openly recruited healthy volunteers. They were clearly told the use of their blood samples before signed informed consent forms and drawn 50 mL blood each by a certified medical staff. All efforts were made to reduce animal suffering and the number of animals used. All animals used in the study received care according to the *Guide for the Care and Use of Laboratory Animals* (Washington DC: National Academy Press, 1996), and was approved by the Animal Administrative Committee of Xi'an Jiaotong University.

### 2.2 Amplification of OECs

Obtaining rabbit PB and BM and from them isolating mononuclear cells (MNCs) refer to our previous report.^4^ Human PB MNCs were isolated in the same way. Rat BM was harvested from the femurs and tibias of male Sprague‐Dawley rats (250‐280 g, supplied by the Laboratory Animal Center of Xi'an Jiaotong University) killed with inhalation of CO_2_. MNCs were plated on 100‐mm culture dishes that had been coated with human fibronectin (for PB OECs) or rat collagen type I (for BM OECs), and were cultured for over 2 weeks in endothelial growth medium‐2 (EGM‐2, Lonza‐BioWhittaker, Walkersville*,* MD, USA) at 37°C, letting the included OECs increased while other cell types disappeared. The medium was changed every other day. Cells were subcultured when they approached 90% confluence. For bilobalide groups, bilobalide (0.1‐10 μmol/L; Sigma‐Aldrich, St Louis, MO, USA) was added into EGM‐2. Bilobalide was dissolved in DMSO and diluted with PBS beforehand. The final concentration of DMSO was below 0.05% (v/v).

### 2.3 OEC characterizing

The LDL uptake and agglutinin binding were indicated by 1,1′‐dioctadecyl‐3,3,3′,3′‐tetramethylindocarbocyanine‐labelled acetylated LDL (Dil‐acLDL, Molecular Probes, Eugene, OR, USA) and FITC‐conjugated lectin from *Ulex europaeus* (FITC‐lectin, Sigma‐Aldrich), respectively.^4^ The existences of CD 144, CD14 and CD45 were determined with FACS.^4^ FITC‐conjugated rabbit polyclonal antibody (pAb) against human CD144 and mouse monoclonal antibody (mAb) against human CD14 were purchased from GenWay (San Diego, CA, USA), and FITC‐mAb against rabbit CD45 from Research Diagnostics (Scottsdale, AZ, USA). The existences of eNOS and caveolin‐1 were confirmed with immunofluorescence staining. Two 1 cm^2^‐areas of cells fixed by paraformaldehyde (2%) were crayon‐circled, one was for eNOS and the other was for caveolin‐1. After permeabilization by 5% goat serum, 0.1% Triton X‐100 and 10 mmol/L glycine, the circled cells were incubated for 1 hour in 10% goat serum at room temperature, and 1 hour in anti‐rabbit eNOS mAb (1:200, BD Biosciences, San Jose, CA, USA) or in anti‐human caveolin‐1 mAb (1:200, Abcam, Cambridge, MA, USA) at 37°C. Then, they were incubated for 1 hour in goat anti‐mouse immunoglobulin conjugated with Texas Red (1:50 dilution, Jackson Immuno Research Laboratories, West Grove, PA, USA) for eNOS or with FITC (1:50, Sigma‐Aldrich) for caveolin‐1 at 37°C, and were incubated for 5 minutes in Hoechst 33258 (100 ng/mL, Sigma‐Aldrich) at room temperature. Each step finished as three washes by PBS containing 10 mmol/L glycine. The fluorescence*‐*stained cells were mounted with ProLong Gold Antifade Reagent (Invitrogen, Carlsbad, CA, USA) before observed. The tube formation test used Matrigel Matrix (BD biosciences).^4^

### 2.4 Mouse femoral artery injury and treatment by OECs or/and bilobalide

Male BALB/c nude mice weighing 34‐41 g (Beijing Vital River Laboratory Animal Technology Co., Ltd, Beijing, China) were anaesthetized by intraperitoneal injection of pentobarbital (50 mg/kg). Transluminal mechanical injury of the femoral artery was performed according to the literature reports.^5^, ^19^, ^20^ Briefly, the left femoral artery and a branch between the rectus femoris and vastus medialis muscles were exposed and looped proximally and distally with silk suture. Transverse arteriotomy was performed in the exposed muscular branch artery, through which a straight spring wire (0.38 mm diameter) was inserted into the femoral artery. The wire was slowly push‐and‐pulled for 4 times and left in place for 1 minute to denude and dilate the artery, then was removed and the suture looped at the proximal portion of the muscular branch artery was secured. Blood flow in the femoral artery was restored by releasing the sutures placed in the proximal and distal femoral portions.

The model mice were divided into 5 groups: PBS, OECs, bilobalide 5 and 10 mg/kg, and OECs + bilobalide (10 mg/kg). For mice in PBS group, PBS (1 mL/kg) was injected through tail vein at 1 hour after endothelial denudation. For OECs groups, bilobalide‐based human OECs (means the cells were obtained through culture in bilobalide‐contained EGM2) were intravenously injected 5 × 10^5^ per mouse. For bilobalide groups, bilobalide was intraperitoneally injected 5 or 10 mg/kg per day for 4 weeks. In addition, extra 3 animals were respectively added to the OECs group and OECs + bilobalide group, which were transfused BrdU‐labelled OECs (5 × 10^5^) and sampled arteries to observe whether the transfused bilobalide‐based human OECs could home to the injured vascular intima. Each of the rest animals was collected blood 0.2 mL at day 5, via unilateral eyeball enucleations under anaesthesia, and 0.7‐1.1 mL at day 28, by cardiac puncture after euthanized by deep anaesthesia, from which MNCs were immediately separated to quantify included OECs (CD 144 positive and CD45 negative) via FACS. Femoral arteries were sampled at day 28 to make 5‐μm thick frozen sections and H&E‐stained. Indices of the neointima formation were analysed with the image analysis system (Q550CW, Leica) as our previous report.^4^ Most importantly, the internal elastic lamina was set as the boundary between intima and media.

### 2.5 5‐bromo‐2′‐deoxyuridine (BrdU) labelling of OECs and BrdU‐positive cells identification

Late‐outgrowth endothelial cells (OCEs) were incubated in BrdU (5 μg/mL; (5 μg/mL, Sigma‐Aldrich)) and FBS (10%)‐contained EBM‐2 for 48 hours before transfusion into the femoral injured mice. BrdU**‐**positive cells were identified by immunohistochemistry. Related details refer to our previous report.^4^

### 2.6 MTT assay

Late‐outgrowth endothelial cells or HUVECs (ATCC, Manassas, VA, USA) were plated on 96‐well dishes (1 × 10^4^ cells/well) and cultured in endothelial basal medium (EBM‐2, Lonza‐BioWhittaker) without serum for 24 hours, and in EBM‐2 with FBS (10%) or VEGF (100 ng/mL, Lonza‐BioWhittaker) for 5 days. For bilobalide groups, bilobalide was added into EBM‐2 at the same time as FBS or VEGF. For *N* ^G^‐nitro‐L‐arginine methyl ester (L‐NAME) groups, L‐NAME (5 μmol/L, Sigma‐Aldrich) was added 30 minutes prior to FBS or VEGF. For 2‐(2‐amino‐3‐methoxyphenyl)‐4H‐benzopyran‐4‐one (PD98059) groups, PD98059 (10 μmol/L, Selleck, Houston, TX, USA) was added 4 hours prior to FBS or VEGF. Cells harvested were incubated for 4 hours in PBS containing MTT (0.5 mg/mL, Sigma‐Aldrich) at 37°C followed by elimination of supernatant and washing twice. To each well was added 200 μL DMSO, followed by shaking for 10 minutes, and detection of the OD by a microplate spectrophotometer (Bio Rad) set to a wavelength of 490 nm.

### 2.7 BrdU assay

Late‐outgrowth endothelial cells were plated on 8‐well chamber slides (3 × 10^4^ cells/well) and underwent incubation in EBM‐2 for 24 hours, in EBM‐2 containing FBS (10%) with BrdU for 48 hours and in that without BrdU for 4 days. In bilobalide group, bilobalide was added into EBM‐2 simultaneously with FBS. Cells harvested were fixed with paraformaldehyde, denatured for 60 minute with HCl (2 mol/L), neutralized for 10 minutes with sodium borate (0.1 mol/L, pH 8.5), and incubated for 60 minutes in anti‐BrdU mAb (2 μg in 1% BSA/well, Sigma‐Aldrich) at 37°C, 60 minutes in FITC‐goat anti‐mouse immunoglobulin and 5 minute in Hoechst. Each step finished as three washes of cells by PBS.

### 2.8 Measuring intracellular nitric oxide, superoxide anion, H_2_O_2_ and hydroxyl radical/peroxynitrite

Nitric oxide, superoxide anion (O_2_^− •^) and hydroxyl radical (OH^•^)/peroxynitrite (ONOO^−^) levels were determined with the fluorescence probes 3‐amino,4‐aminomethyl‐2′,7′‐difluorescein diacetate (DAF‐FM DA; Beyotime Institute of Biotechnology, Haimen, China), dihydroethidium (DHE, Beyotime Institute of Biotechnology) and hydroxyphenyl fluorescein (HPF; Cell Technology Inc, Fremont, CA, USA), respectively. OECs plated in 96‐well dishes (2 × 10^4^ cells/well) were incubated in EBM‐2 for 24 hours then in EBM‐2 with DAF‐FM DA (5 μmol/L) or DHE (3 μmol/L) for 50 minutes, or in EBM‐2 with HPF (5 μmol/L) for 30 minutes. After washed extracellular dye, the dye‐loaded cells were incubated in EBM‐2 with (for nitric oxide) or without (for O_2_^−•^ or OH^•^/ONOO^−^) L‐arginine (1.15 mmol/L, Sigma‐Aldrich) for 20 minutes. Next, VEGF (100 ng/mL) was added into the media of proliferative but not quiescent cell groups. For bilobalide‐treatment groups, bilobalide was added immediately after VEGF into the media. For bilobalide‐pre‐treatment groups, bilobalide was added 24 hours prior to VEGF (means before dye‐load). At the designed time‐points, cell fluorescence intensity was measured using a fluorescence spectrophotometer. The excitation and emission wavelengths were, respectively, set at (nm): 495 and 515 for nitric oxide, 535 and 610 for O_2_^−•^ and 488 and 515 for OH^•^/ONOO^−^. A well of OECs without dye was set as the blank control.

Intracellular H_2_O_2_ was quantified with a H_2_O_2_ assay kit (Beyotime Institute of Biotechnology). The principle of this kit refers to the literature.^21^ All operations were carried out according to the manufacturer's instruction. Briefly, treatments of starved OECs with VEGF and bilobalide are the same as stated above. At 5 minute after stimulation by VEGF, cells were collected and lysed in cold lysis buffer solution (100 μL/10^6^ cells) from the kit. The supernatants were gained through high‐speed freeze centrifugation and added into the test tubes containing test solutions (at room temperature) to measure the absorbance at 560 nm using a microplate reader. The H_2_O_2_ level was calculated according to a standard concentration curve originated from standard solutions.

### 2.9 Transfection of siRNA

eNOS‐, iNOS‐ or negative control siRNA (Santa Cruz Biotechnology, Santa Cruz, CA, USA), at a final concentration of 100 nmol/L, was transfected into OECs using siRNA transfection reagent (Santa Cruz Biotechnology) in transfection medium (Santa Cruz Biotechnology) for 6 hours according to the manufacturer's instructions. Transfected cells were washed with PBS and incubated in EBM‐2 for 24 hours before confirmed knock‐down of the target proteins by Western blotting or started experiments.

### 2.10 Migration assay

The modified Boyden chamber assay 22 was used. Briefly, transwell inserts (Costar Transwell membrane, 6.5 mm diameter, 12.0 μm pore size) were placed in 24‐well dishes. Rabbit PB OECs were plated on the upper wells (5 × 10^4^ cells/well) to culture for 24 hours in EBM‐2 containing FBS (5%) and stromal cell‐derived factor‐1α (SDF‐1α, 100 ng/mL; Sigma‐Aldrich). Adding bilobalide and L‐NAME into the media of related groups refers to the above description. Cells that migrated to the underside of the membrane were Hoechst‐stained for counting.

### 2.11 Western blotting

Cells were lysed in cold extract buffer (50 mmol/L Tris‐HCl, pH 7.4, 1 mmol/L DTT, 1 mmol/L EDTA). Protein concentration was measured with Bradford method. Equal amounts of protein (50 μg) were separated by electrophoresis in a 7.5% polyacrylamide‐SDS gel and electrophoretically transferred to a nitrocellulose membrane of 0.45 μm pore size in glycine‐methanol buffer. The membrane was blocked in Odyssey blocking buffer (LI‐COR Biosciences, Lincoln, NE, USA) for 1 hour at room temperature, and then incubated overnight at 4°C in rabbit anti‐human pAb against ERK1/2 (1:250 dilution; Biovision, San Francisco, CA, USA) or against phosphorylated ERK1/2 (P‐ERK1/2, Thr‐202/Tyr‐204, 1:2000; Santa Cruz Biotechnology), or in rabbit anti‐human mAb against eNOS (1:200, BD Biosciences) or against P‐eNOS (at Ser‐1177 or Thr‐495, 1:1000; Cell Signalling Technology, Beverly, MA, USA). After incubated for 1 hour in IR Dye 800‐goat anti‐rabbit or mouse immunoglobulin (LI‐COR Biosciences), bands were visualized with Odyssey Imager and quantified with NIH Image J software. Equal protein loading was checked by detection of GAPDH protein using anti‐rabbit GAPDH mAb (1 μg/mL; LifeSpan Biosciences, Seattle, WA, USA).

### 2.12 Statistics

Data are presented as mean ± SD for at least three samples or three repeated individual experiments per group. Tukey's post hoc test after ANOVA used GraphPad Prism v5.0 software. *P* value <.05 was considered significant.

## 3 RESULTS

### 3.1 Bilobalide enhances OEC number increase

**Figure 1:**
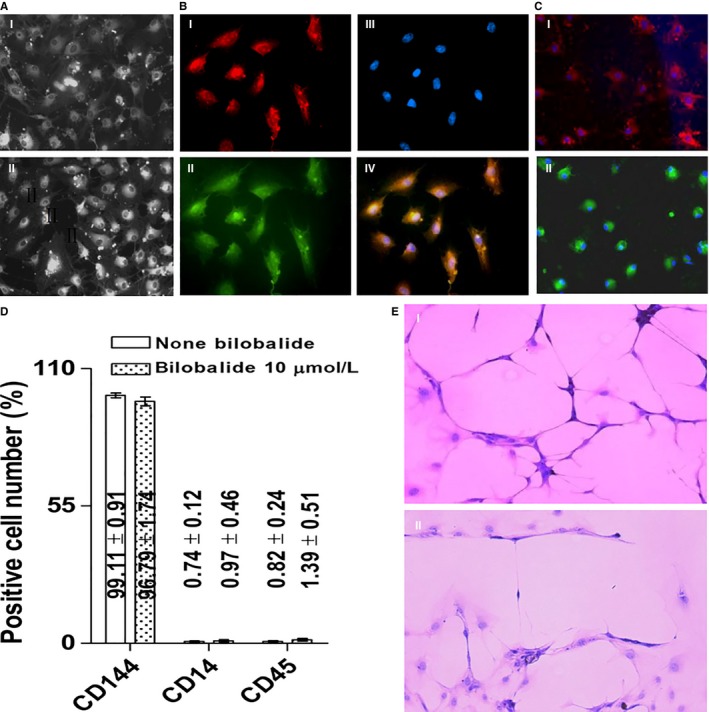
Characterization of late‐outgrowth endothelial cells (OECs). Rabbit peripheral blood (PB) mononuclear cells (MNCs) were cultured in endothelial growth medium (EGM‐2) for 4 weeks to expand the included OECs. Bilobalide (0.1‐10 μmol/L) was added into EGM‐2 to improve the expansion. The harvest cells with the background of 10 μmol/L bilobalide were assessed. A, Shapes seen under light microscope (×200). (I) None bilobalide; (II) 10 μmol/L bilobalide. B, Taking in LDL and binding lectin (×400). (I) Dil‐acLDL; (II) FITC‐lectin; (III) Hoechst (for the nucleus); (IV) overlap. C, Immunofluorescent staining of eNOS (I) and caveolin‐1 (II) (×200). D, CD144^+^−, CD14^+^−, and CD45^+^ cell percentages analysed by FACS (n = 4 per group). E, Forming tube‐like structures on the Matrigel Matrix‐treated dishes (×100). (I) None bilobalide; (II) 10 μmol/L bilobalide

Mononuclear cells (MNC) separated from rabbit PB and BM and human PB were, respectively, seeded 1 × 10^7^ and cultured in EGM‐2 to expand the included OECs. Four weeks later, as shown in Table 1, in control groups, the numbers of OECs harvested were 2.63 × 10^5^, 2.71 × 10^6^ and 2.58 × 10^5,^ respectively; bilobalide, at 0.3‐10 μmol/L, increased the OEC harvests. The increase rates of rabbit PB‐ and BM‐ and human PB OEC harvests, by 10 μmol/L bilobalide, are 234, 220 and 230%, respectively. Bilobalide did not alter the OEC characteristics. For example, the PB OECs that were amplified in the presence of 10 μmol/L bilobalide have cobblestone shape when they are at adhesion state (Figure 1A), can take in LDL and bind agglutinin (Figure 1B), express CD 144, eNOS and caveolin‐1 but not CD 14 and CD 45 (Figure 1C,D), and can form tube‐like structures on a matrix (Figure 1E), according with the OEC characteristics described previously.^4^

**Table 1:** Numbers of OECs harvested after in vitro expansion

Bilobalide (μmol/L)	Rabbit PB‐OECs		Rabbit BM‐OECs		Human PB‐OECs	
	n	Count (×10^5^)	n	Count (×10^6^)	n	Count (×10^5^)
0 (Control)	6	2.632 ± 0.856	7	2.712 ± 0.528	5	2.576 ± 0.539
0.1	7	3.211 ± 0.683	7	3.062 ± 0.309	5	3.092 ± 0.599
0.3	7	4.474 ± 0.654*	7	4.870 ± 0.745**	5	4.392 ± 0.401*
1	6	5.662 ± 0.872**	7	6.383 ± 0.919**	5	5.718 ± 0.369**
3	7	7.657 ± 1.208**	7	8.097 ± 0.758**	5	7.208 ± 0.523**
10	5	8.800 ± 1.275**	7	8.676 ± 1.159**	5	8.492 ± 0.915**

Mononuclear cells (MNCs) form rabbit and human peripheral blood (PB) and rabbit bone marrow (BM) were, respectively, seeded 1 × 10^7^ and cultured in endothelial growth medium‐2 (EGM‐2) for 4 weeks to expand the included late‐outgrowth endothelial cells (OECs). Bilobalide was added as the indicated concentration into EGM‐2.

*P < .05 and **P < .01 vs control.

### 3.2 Bilobalide‐based OECs but not bilobalide reduce the intimal hyperplasia in femoral after injury

**Figure 2:**
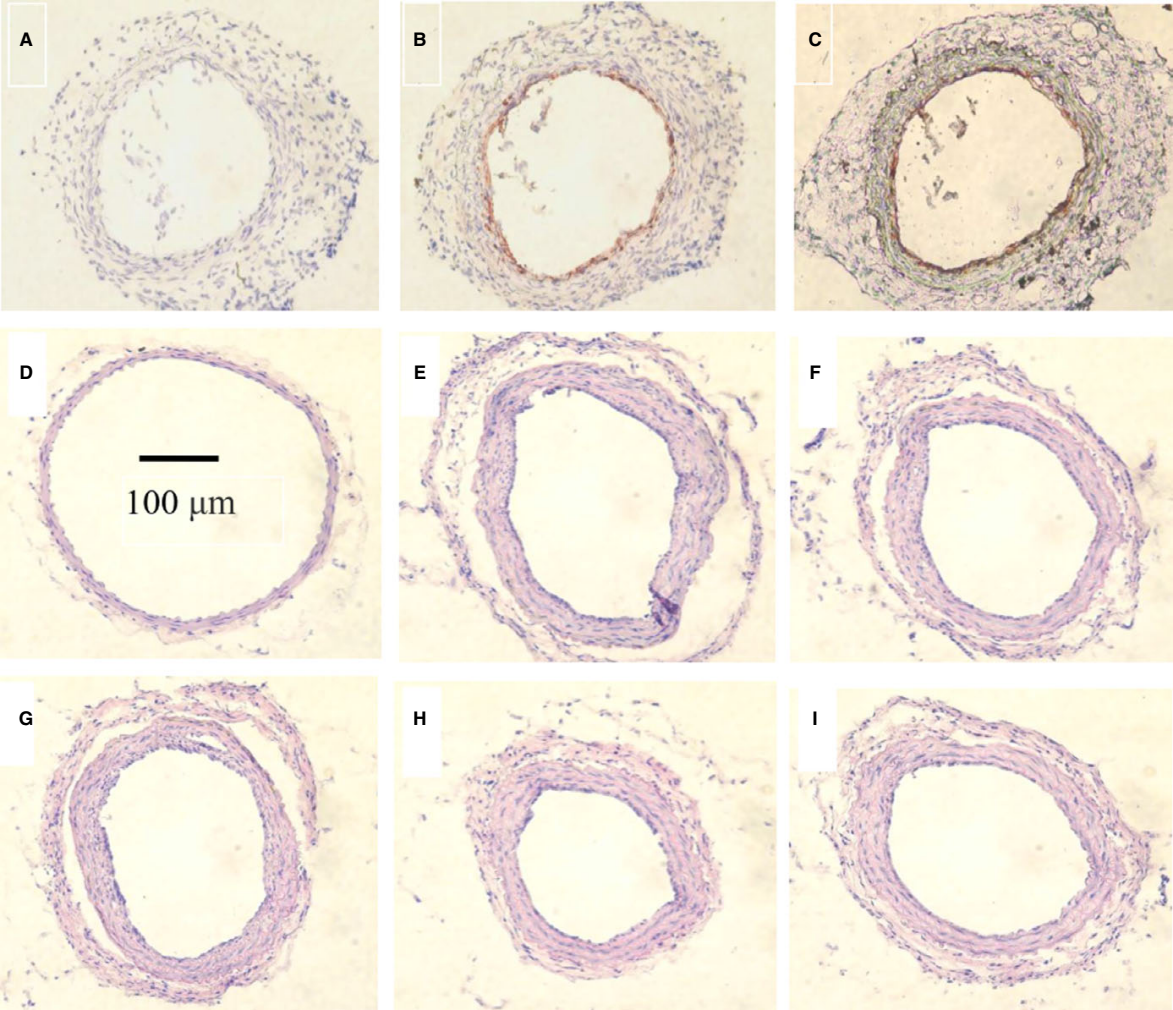
Representative micrographs of athymia nude mouse femoral artery after endothelium‐injury. A‐C, Immunohistochemistry staining. BrdU‐positive cells (brown) are seen on the injured arterial intima of late‐outgrowth endothelial cells (OECs) group (B) and OECs + bilobalide group (C) but not PBS group (A) at day 4 after BrdU‐labelled bilobalide human OECs transfused. D‐I, H&E‐stained sections of the femoral at day 28 after injury. D, Normal (no injury). Intravenous injection of PBS (E), and intraperitoneal injection of bilobalide 5 (F) or 10 mg/kg (G) per day for 4 weeks did not reduce neointimal hyperplasia. Transfusion of bilobalide‐based human OECs 5 × 10^5^ (H) and the OEC transfusion + bilobalide administration (10 mg/kg per day) (I) reduced the neointimal hyperplasia

Athymia nude mouse femoral artery injury model was used to evaluate the effectiveness of bilobalide and bilobalide‐based OECs in vivo. Average 1.2 × 10^6^ MNCs were obtained from 1 mL of blood sample. From the fresh MNCs of all groups (at day 5 and 28), no OEC (CD144^+^ and CD45^−^ cell) was detected. However, on the injured arterial intima of mouse in OECs group and OECs + bilobalide group, BrdU‐positive cells could be seen at day 4 after BrdU‐labelled bilobalide human OECs transfused, illustrating the OECs homing to injury site (Figure 2A‐C). And as shown in Figure 2D‐I and Table 2, in PBS (model) group, the intima area of the femoral artery increased significantly at day 28 after injury while the media area remained unchanged, and thus the lumen area decreased significantly. Administrations of bilobalide 5 and 10 mg/kg/day for 4 weeks both did not alter the intima area increase and lumen area decrease. The intima area increase and lumen area decrease were relieved by transfusion of bilobalide‐based human OECs and the scheme of OEC transfusion + bilobalide administration. Additionally, although there is no difference in the media area between injured and uninjured arteries, most of injured arteries’ thickness increased and the elastic laminas were bent (including OECs group), suggesting the vasoconstriction induced by endothelial injury is lasting. Although there is no difference in the intima area and media area between the OECs and OECs + bilobalide groups, the lumen area of OECs + bilobalide group is larger than that of OECs alone group, conforming bilobalide's vasodilating effect.

**Table 2:** Intima, media and lumen areas of mice femoral arteries after endothelial injury

Groups	LA (μm^2^)	IA (μm^2^)	MA (μm^2^)	IA/MA (%)
No injury	61 418 ± 8462**	2612 ± 207**	18 281 ± 3012	14.7 ± 6.3**
PBS	32 548 ± 6142	21 654 ± 3736	24 351 ± 5016	94.4 ± 6.2
Bilobalide (5 mg/kg/day)	30 146 ± 4026	20 714 ± 2148	21 426 ± 3024	96.2 ± 7.7
Bilobalide (10 mg/kg/day)	32 064 ± 5125	22 204 ± 3218	23 125 ± 4105	95.8 ± 11.2
OECs	42 275 ± 7063**	3232 ± 147**	19 619 ± 4121	16.5 ± 4.2**
OECs + Bilobalide (10 mg/kg/day)	50 845 ± 5747** ^,^ *	3072 ± 188**	20 372 ± 2025	15.1 ± 6.6**

The right femoral artery of the model mouse was used as no injury artery. IA, intima area; LA, lumen area; MA, media area; OEC, late‐outgrowth endothelial cells.

Data are presented as means ± SD, n = 5 in each group.

**P < .01 vs PBS; * *p* < .05 vs OECs.

### 3.3 Bilobalide promotes OEC proliferation and increases intracellular nitric oxide level

**Figure 3:**
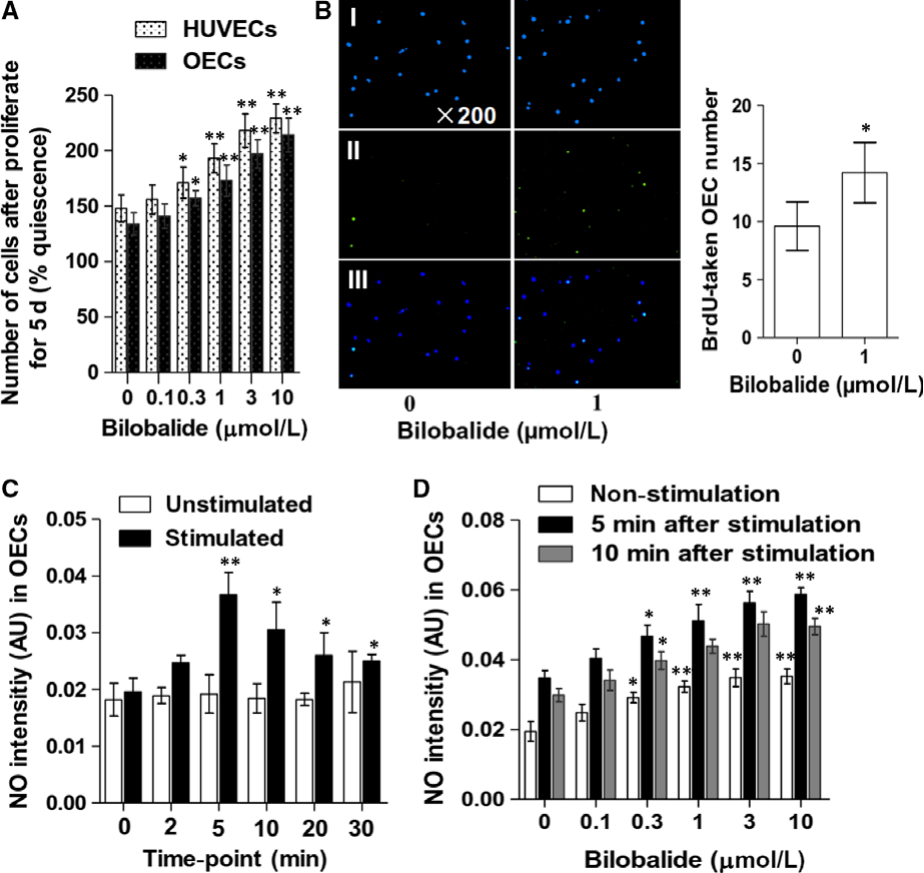
Effects of bilobalide on late‐outgrowth endothelial cells (OEC) proliferation and intracellular nitric oxide level. Rabbit PB OECs were stimulated with FBS (10%) (A,B) or VEGF (100 ng/mL) (C,D) to induce proliferating. In bilobalide groups, bilobalide was added simultaneously with FBS or VEGF. A, Cell viability measurement by MTT assay. Quiescence, unstimulated. B, BrdU assay. Left, representative images: (I), Hoechst; (II), BrdU antibody; (III), overlap. Right, statistics: each sample was randomly chosen 3 visual fields (×200) to count the BrdU‐taken OECs. OEC nitric oxide was quantified with DAF‐FM DA. C, Time course of nitric oxide level change in OECs. D, Effects of bilobalide on OEC nitric oxide level. n = 6 (A) or 5 (B,D) or 4 (C) per group. *P < .05 and **P < .01 vs control (0 μmol/L bilobalide)

BrdU incorporation assay and cell viability measurement by MTT assay, respectively, are often used direct and indirect methods for attesting cell proliferation. In this study, MTT assay showed that HUVECs and rabbit PB OECs increased their numbers to 148 ± 12% and 134 ± 10%, respectively, after cultured for 5 days in FBS (10%)‐contained EBM‐2; bilobalide concentration (0.3‐10 μmol/L) dependently promoted the two cell types’ growth (Figure 3A). Similarly, BrdU assay verified that bilobalide (1 μmol/L) accelerates the FBS‐induced OEC proliferation (Figure 3B).

Rabbit PB OECs were loaded with DAF‐FM DA to indicate intracellular nitric oxide and then were incubated in EBM‐2 without and with VEGF (100 ng/mL) to keep quiescent and to induce proliferating, respectively. There were insignificant differences among the nitric oxide levels detected at 0, 2, 5, 10, 20 and 30 minutes in the quiescent cells. In the proliferating cells, the nitric oxide levels at 5, 10, 20 and 30 minutes, especially at 5 and 10 minutes, were significantly higher than that at 0 minutes (Figure 3C). As shown in Figure 3D, bilobalide, at 0.3‐10 μmol/L, increased the nitric oxide levels in the quiescent OECs (detected at 5 minutes) and in the proliferating OECs (detected at 5 and 10 minutes). Such rapid effect implies that bilobalide might increase the activity of any NOS type in OECs.

### 3.4 Nitric oxide mediates bilobalide‐promoting OEC proliferation

**Figure 4:**
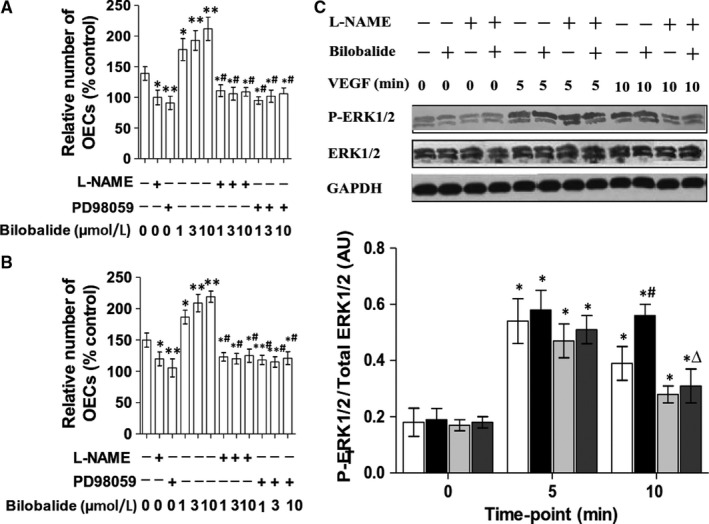
Effects of nitric oxide synthesis inhibition on bilobalide‐promoting OEC's proliferation and ERK1/2 activation. Rabbit PB OECs were treated with L‐NAME (5 μmol/L) or PD98059 (10 μmol/L) for 30 min before exposed to FBS (A) or VEGF (B) and tested viability by MTT assay. Rat BM OECs were pre‐treated with L‐NAME then exposed to VEGF and detected P‐ERK1/2 at the indicated time‐points (C). In bilobalide groups, bilobalide (1 μmol/L) was added at the same time as FBS (10%) or VEGF (100 ng/mL). (A,B) n = 4 per group. *P < .05 and **P < .01 vs L‐NAME (−) + PD98059 (−) + bilobalide (−); ^#^ *P* < .01 vs L‐NAME (−) + PD98059 (−) + bilobalide (+). (C) Upper, representative Western blotting bands; Lower, statistics: n = 4 per group. *P < .01 vs 0 min; ^#^ *P* < .01 vs L‐NAME (−) + bilobalide (−); ^▵^ *P* < .01 vs L‐NAME (−) + bilobalide (+)

MTT assay showed that pre‐treatments by L‐NAME (5 μmol/L), a NOS inhibitor, and PD98059 (10 μmol/L), a MAPK kinase inhibitor, both slow FBS‐ and VEGF‐induced proliferations of rabbit PB OECs, and abolish bilobalide (1‐10 μmol/L)'s increase effect on the proliferations (Figure 4A,B). Another experiment using rat BM OECs showed that VEGF‐induced ERK1/2 phosphorylation, more P‐ERK1/2 was detected at 5 minutes than that at 10 minutes; bilobalide (1 μmol/L) did not affect the P‐ERK1/2 at 5 minutes, but increased that at 10 minutes; L‐NAME pre‐treatment did not alter the VEGF‐based P‐ERK1/2, but abolished the increase in bilobalide of P‐ERK1/2 (Figure 4C).

### 3.5 Bilobalide promotes OEC eNOS activation

**Figure 5:**
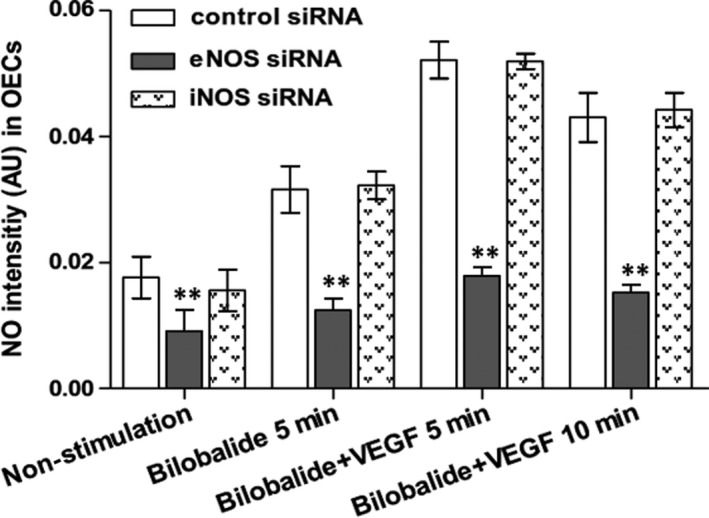
Effects of eNOS‐ and iNOS knock‐downs on nitric oxide level in OECs. Rat BM OECs were decreased eNOS‐ and iNOS expression by the respective siRNA. Non‐stimulation means cells are in EBM‐2 without serum, VEGF (100 ng/mL) and bilobalide (1 μmol/L). n = 3 per group. **P < .01 vs control siRNA

**Figure 6:**
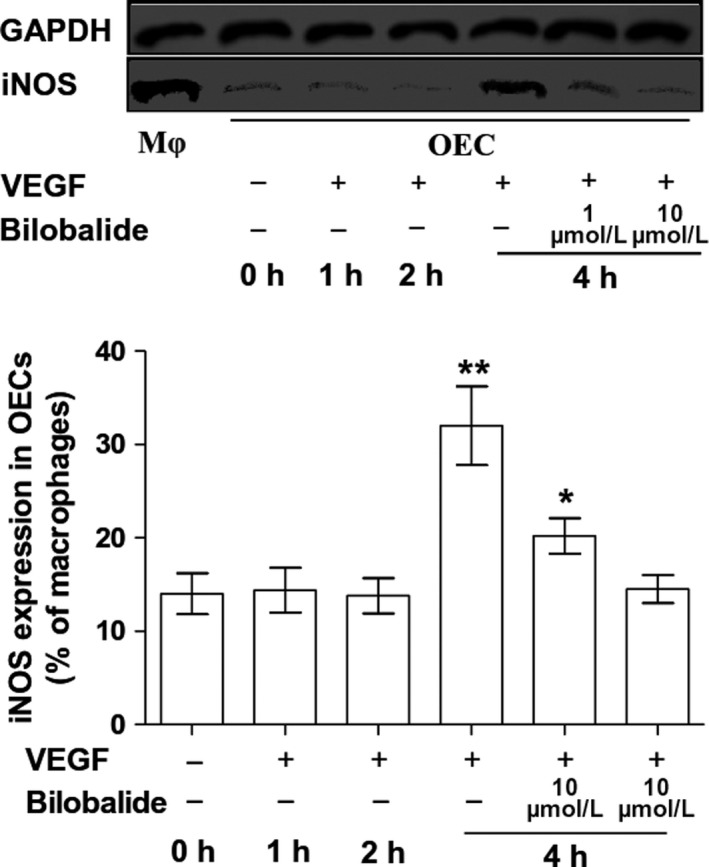
Effects of bilobalide on rat BM OEC iNOS expression. Upper, representative Western blotting bands (Mφ, rat macrophages). Bottom, statistics: n = 3 per group. *P < .05 and **P < .01 vs 0 h. VEGF concentration was 100 ng/mL

**Figure 7:**
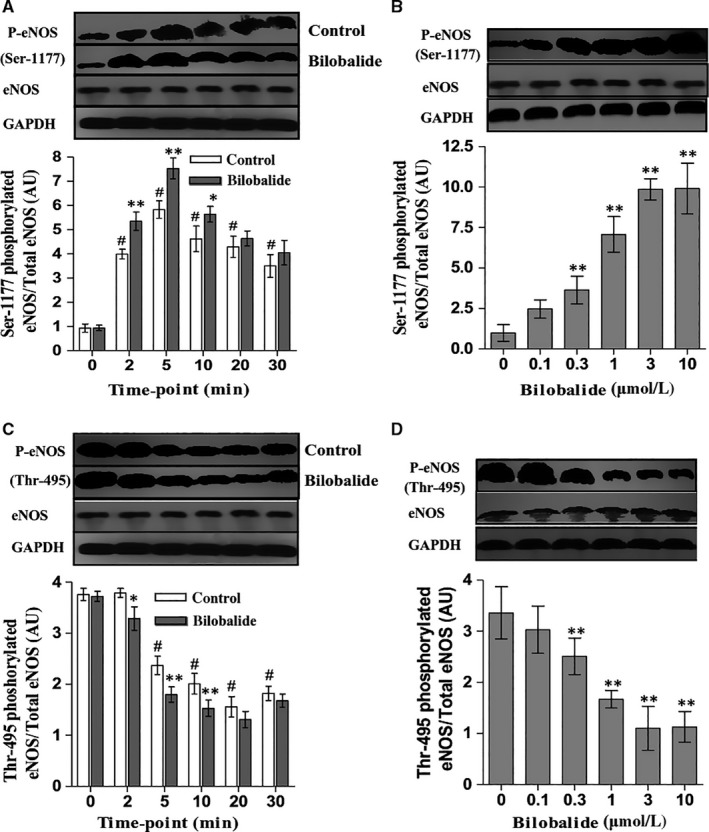
Effects of bilobalide on late‐outgrowth endothelial cells (OEC) eNOS activation. Serum‐starved rat BM OECs were exposed to FBS (10%). In bilobalide groups, bilobalide was added at the same time as FBS. A,B, phosphorylated eNOS at Ser‐1177. C,D, phosphorylated eNOS at Thr‐495. In (A,C), bilobalide concentration was fixed at 1 μmol/L. The detection time was fixed at 5 (B) or 20 min (D). n = 4‐5 per group. *P < .05 and **P < .01 vs control (0 μmol/L bilobalide); #*P* < .01 vs 0 min

Late‐outgrowth endothelial cells constitutively express eNOS and iNOS.^10^ We used siRNA to decrease rat BM OECs expressing eNOS and iNOS; the decrease rates were 62.4% and 67.2%, respectively. The eNOS knock‐down decreased the nitric oxide level in the quiescent OECs by 48.3%, decreased the bilobalide (1 μmol/L)‐based OEC nitric oxide by 60.8% (at 5 minutes after added bilobalide), and decreased the bilobalide plus VEGF (100 ng/mL)‐based OEC nitric oxide by 65.6% at 5 minutes and 64.7% at 10 minutes. The iNOS knock‐down decreased 7.1% of nitric oxide in the quiescent OECs, but did not affect the bilobalide alone‐ and plus VEGF‐based intracellular nitric oxide (Figure 5). Bilobalide (1 and 10 μmol/L) did not affect rat OECs’ eNOS protein mass, data detected at 0, 1, 3, 6, 12 and 24 hours (Figure S2). Compared with macrophages, OECs have weaker basal iNOS expression. VEGF increases while bilobalide decreases OEC iNOS expression; the effects appeared in 4 hours (Figure 6). In serum‐free EBM‐2, the OEC eNOS was strongly phosphorylated at Thr‐495 but only weakly at Ser‐1177. There were no differences among the data gathered at 0, 2, 5, 10, 20, 30 minutes (Figure S3); addition of FBS (10%) increased P‐Ser‐1177, peaking at 5 minutes of 30‐minutes discontinuous detections (Figure 7A), and decreased P‐Thr‐495, the trough appeared at 20 minutes (Figure 7C); bilobalide enhanced both the Ser‐1177 phosphorylation and Thr‐495 dephosphorylation in a concentration‐dependent manner (0.1‐3 μmol/L) (Figure 7B,D).

### 3.6 Quenching reactive oxygen species contributes to bilobalide enhancing OEC nitric oxide

**Figure 8:**
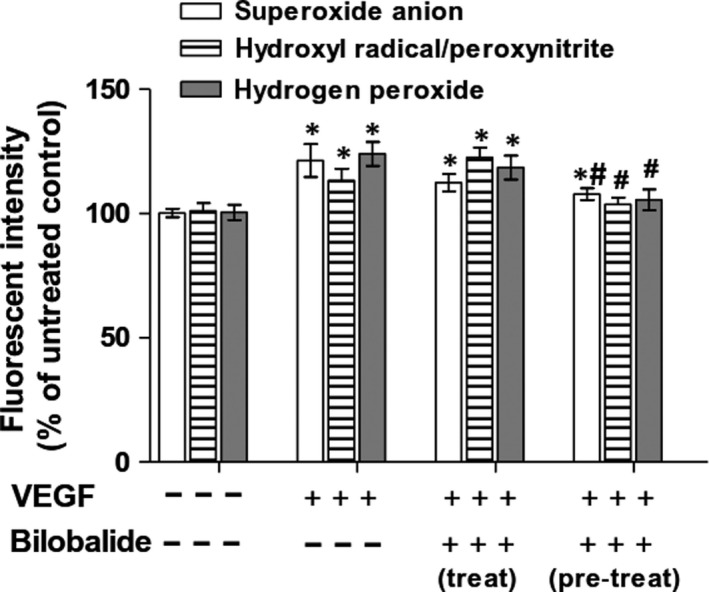
Effect of bilobalide on hydroxyl radical/peroxynitrite, superoxide anion and hydrogen peroxide in proliferating late‐outgrowth endothelial cells (OECs). Rabit PB OECs were stimulated with VEGF to induce proliferating. Data were detected at 5 min after stimulation. Bilobalide treatment means adding bilobalide (1 μmol/L) immediately after VEGF, pre‐treatment means adding bilobalide 24 h prior to VEGF. Intracellular hydroxyl radical/peroxynitrite was determined by hydroxyphenyl fluorescein, and superoxide anion by dihydroethidium, hydrogen peroxide by a hydrogen peroxide kit. n = 3 per group. *P < .05 or .01 vs VEGF (−); #*P* < .01 vs VEGF (+) + bilobalide (−)

Nitric oxide reacts with O_2_^− •^ forming ONOO^−^.^23^ Bilobalide has antioxidant potential in a variety cell types because of promoting antioxidase expression.^18^, ^19^, ^24^, ^25^ We used rabbit PB OECs to dissect whether bilobalide quenching reactive oxygen species (ROS) plays a role in its enhancing nitric oxide level in proliferating OECs. As shown in Figure 8, stimulation of OECs with VEGF (100 ng/mL) caused intracellular OH^•^/ONOO^−^ as well as O_2_^− •^ and H_2_O_2_ increased (data detected at 5 minutes). Bilobalide (1 μmol/L) treatment (added immediately after VEGF) did not eliminate the increase by VEGF of O_2_^− •^ and H_2_O_2_, and even enhanced OH^•^/ONOO^−^ slightly. However, bilobalide pre‐treatment (added 24 hours prior to VEGF) significantly eliminated the VEGF‐induced excess production of OH^•^/ONOO^−^, O_2_^− •^ and H_2_O_2_. The results imply that bilobalide might reduce nitric oxide depletion by scavenge ROS in OECs.

### 3.7 Bilobalide promotes OEC migration

**Figure 9:**
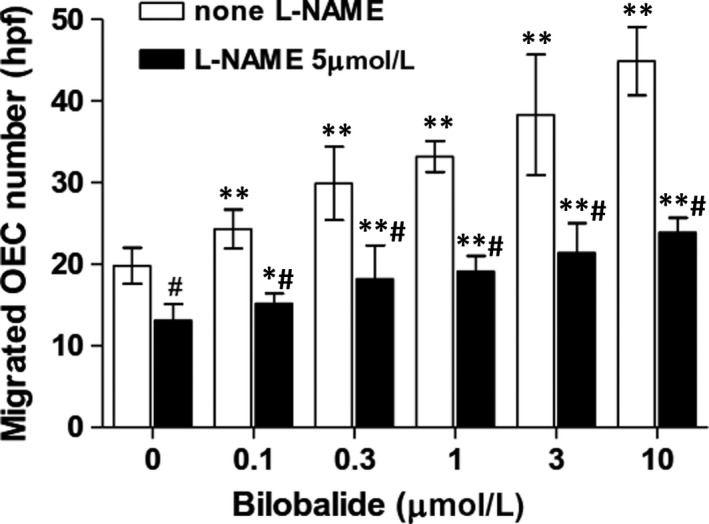
Effect of bilobalide on migration of rabbit PB OECs. The modified Boyden chamber assay was used. Additions of bilobalide and L‐NAME refer to Figure 3. For each sample, migrated cells in 3 randomly chosen visual fields (×100) were counted and averaged. n = 6‐7 per group. *P < .05 and **P < .01 vs 0 μmol/L bilobalide; #*P* < .01 vs none L‐NAME

As shown in Figure 9, some of the OECs that had been plated on the upper well of the transwell insert migrated to the underside of transwell membrane within 24 hours of incubation. L‐NAME (5 μmol/L) pre‐treatment decreased the migration by 33.8%. Bilobalide concentration (0.1‐10 μmol/L)‐dependently increased the OEC migration, at 10 μmol/L, its increase rate was 126.8%. The effect was decreased by L‐NAME too: L‐NAME (5 μmol/L) pre‐treatment decreased the 10 μmol/L bilobalide‐based OEC migration by 46.8%.

## 4 DISCUSSION

Late‐outgrowth endothelial cells (OCE) in circulation are rare, estimated to only 0.05‐0.2 cells per mL blood.^3^ Thus, to use OECs for therapy even to see the cells, an expansion in vitro will be necessary to obtain a sufficient number. The major innovation of the present study is verified that bilobalide enhances OEC number amplification in vitro, which is supported by the following findings: (i) bilobalide promotes OEC proliferation and migration thus enhances OECs amplification; (ii) nitric oxide plays a partial role in OEC proliferation and migration; (iii) bilobalide increases nitric oxide level to benefit ERK1/2 activation in activated OECs; (iv) bilobalide promotes eNOS activation to increase nitric oxide production and promotes ROS scavenging to reduce nitric oxide depletion in activated OECs. (v) Transfusion of sufficient numbers of bilobalide‐based OECs soon after vascular injury reduces the intimal hyperplasia.

Because of scarcity and lacking specific markers, OECs are difficult to distinguish directly from other cell types. MNCs originally refer to any blood cell having a round nucleus, including endothelial progenitor cells. Recently, analogous cells in BM are also called MNCs. The Ficoll solution may separate MNCs from polymorphonuclear cells (such as neutrophils and eosinophils) and erythrocytes. OECs can be obtained by culturing PB‐ or BM MNCs for over 2 weeks in the medium that is suitable for EC growth. During the period of culturing, OECs increase while other cell types including early‐outgrowth endothelial progenitor cells disappear.^1^, ^4^ In the present study, to explore whether bilobalide enhances the OEC amplification, we expanded OECs that included in MNCs for 4 weeks; to investigate how bilobalide enhances the OEC amplification, we used OECs harvested after 2‐week culture of MNCs. We mainly used rabbit‐ and human OECs. Some experiments used rat OECs because of the reactivity of available antibodies.

EGM‐2 is an often used culture medium for OECs and is composed of EBM‐2, supplements including VEGF, IGF, EGF, FGF‐B, ascorbic acid, heparin, antibiotics, and FBS which contains a variety of growth factors, hormones and chemokines. We confirmed firstly that in EGM‐2 bilobalide accelerates the OEC amplification. In the following mechanism dissection experiments, to control conditions, we used EBM‐2 with or without certain supplements.

Our previous study has demonstrated that OECs directly participate in the re‐endothelialization and by a paracrine pathway inhibit the migration and proliferation of vascular smooth muscle cells; transfusion of sufficient autologous OECs soon after vascular injury may reduce neointima formation.^4^ In this study, to evaluate the effectiveness of bilobalide‐based OECs in vivo, we injected bilobalide‐based human PB OECs into the femoral artery injured athymia nude mouse. The cell number (5 × 10^5^) refers to the literature.^3^ The reduction in neointima hyperplasia conformed activities of the transfused cells directly. The same animal model was used to test the effect of bilobalide on OECs in vivo. Our preparative experiment had shown that intraperitoneal injection of bilobalide 5 mg/kg could lower mouse blood pressure and dilate mesenteric vasculature. However, injections of bilobalide 5 and 10 mg/kg/day for 4 weeks both did not reduce vascular intima hyperplasia, according to the failure in detection of OECs from blood samples. This does not mean bilobalide cannot enhance OEC number increase in vito because the situations cannot be excluded as follows: (i) the basal OEC number is very low and identification of the cell type is some difficult; (ii) after vascular injury, re‐endothelialization at early stage is important, which needs a large number of OECs; (iii) neonatal OECs might be used by other vascular parts in the body; (iv) bilobalide dosages used below need. Additionally, our study suggests that OECs transfusion + bilobalide administration is superior to OECs transfusion alone because of the vasorelaxation by bilobalide.

Proliferation and migration both contribute to cell number increase. Cell migration facilitates proliferation because it may look for or give a place for the neonatal cells. Our experiments used FBS and VEGF as proliferation inducers and FBS plus extra SDF‐1α as the migration inducer. The present results reveal that bilobalide promotes OEC proliferation and migration.

Nitric oxide plays roles in VEGF‐ and TGFβ1‐inducing EC proliferation and migration 26, 28 and mediates SDF‐1α‐inducing EC migration^29^; OECs have eNOS and can proliferate and migrate while early‐outgrowth endothelial progenitor cells lack eNOS and do not proliferate.^9^, ^10^ Our present data reveal that nitric oxide participates in OEC proliferation and migration but is not indispensable; bilobalide increases nitric oxide level in activated OECs.

Activation of mitogen‐activated protein kinase ERK1/2 plays an important role in cell migration and proliferation. Our study shows that NOS inhibition by L‐NAME and MAPK kinase inhibition by PD98059 both slow FBS‐ and VEGF‐induced proliferations of OECs, and abolish bilobalide (1‐10 μmol/L)'s increase effect on the proliferations, suggesting nitric oxide signalling and MAPK pathway both mediate bilobalide‐promoting OECs proliferating. Further experiment shows that intracellular P‐ERK1/2 as well as nitric oxide increases at 5 minutes after OECs exposure to VEGF, and pre‐treatment by L‐NAME does not abolish the increment of P‐ERK1/2, suggesting up to this time the nitric oxide production and ERK1/2 activation are mutually independent. Bilobalide increases the P‐ERK1/2 at 10 minutes, which is abolished by the L‐NAME pre‐treatment, suggesting nitric oxide mediates bilobalide‐promoting ERK1/2 activation. The case is similar to previous reports that eNOS/NO pathway partly mediates VEGF‐, PGE_2_‐, and Korean red ginseng water extract evoking ERK1/2 activation in ECs.^27^, ^30^, ^31^

Late‐outgrowth endothelial cells have eNOS and iNOS.^10^ Bilobalide decreases iNOS expression in some cell types.^17^, ^19^ The effect of bilobalide on eNOS remains unclear so far. Our present data excluded bilobalide increasing OEC's eNOS‐ and iNOS expression. The eNOS is regulated activity by phosphorylation at multiple sites. Two most thoroughly studied sites are activation site Ser‐1177 and inhibitory site Thr‐495.^32^ Multiple stimuli such as growth factors and fluid shear stress activate eNOS by Ser‐1177 phosphorylation^33^, ^36^; bradykinin and H_2_O_2_ activate eNOS by both Ser‐1177 phosphorylation and Thr‐495 dephosphorylation.^37^, ^38^ Our study found that bilobalide enhances the FBS‐induced OEC eNOS Ser‐1177 phosphorylation and Thr‐495 dephosphorylation.

Reactive oxygen species (ROS) such as O_2_^− •^, H_2_O_2_ and OH^•^ are constantly generated and eliminated in a cell.^39^ They are mainly scavenged by antioxidases. Besides converting into H_2_O_2_ and OH^•^, O_2_^− •^ may react with nitric oxide to form ONOO^−^, degrading nitric oxide.^23^ Certainly, ONOO^−^and H_2_O_2_ may partly convert into OH^•^. Bilobalide has antioxidant potential through promoting antioxidase expression in a variety of cell types.^18^, ^19^, ^24^, ^25^ We failed to get a special reagent for indication of intracellular ONOO^−^ but HPF, a fluorescence probe reactive to OH^•^ and ONOO^−^. Our present data showed that bilobalide pre‐treatment limited the increase of OH^•^/ONOO^−^, O_2_^− •^ and H_2_O_2_ in proliferating OECs, implying bilobalide scavenges ROS to reduce nitric oxide depletion by O_2_^− •^ in activated OECs.

In summary, the present study demonstrates that nitric oxide plays a role in OEC proliferation and migration; bilobalide increases nitric oxide production and prolongs the lifespan of available nitric oxide to enhance OEC growth. The findings may facilitate in vitro expansion of OECs, which may simplify the preparation of OECs for the autologous‐OECs‐transfusion therapy.

## CONFLICT OF INTEREST

The authors confirm that there are no conflict of interest.

## AUTHOR CONTRIBUTIONS

S. Liu designed the study. X. Hou, L. Chen, H. Hu and Q. Sun performed experiments. F. Zhao detected the levels of nitric oxide, OH^•^/ONOO^−^, O_2_^− •^ and H_2_O_2._ C. Liu contributed to data analysis. S. Liu and X. Hou wrote the manuscript. All authors have seen and approved the final version of the manuscript.
