# Supplementary material for: Enhancing amplification of late‐outgrowth endothelial cells by bilobalide
Source: J Cell Mol Med. 2018 Mar 22;22(7):3340–52. doi: 10.1111/jcmm.13609 (PMC6010852; doi:10.1111/jcmm.13609)
Supplement: Supplementary file 1 [file JCMM-22-3340-s001.doc]

**Supplement figures**

**
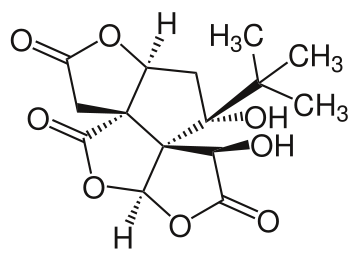
**

**Fig. S1** Chemical structure of bilobalide

**eNOS**

**GADPH**

**Bilobalide**

**1 μM**

**10 μM**

**0**

**1**

**3**

**6**

**12**

**24 (h)**


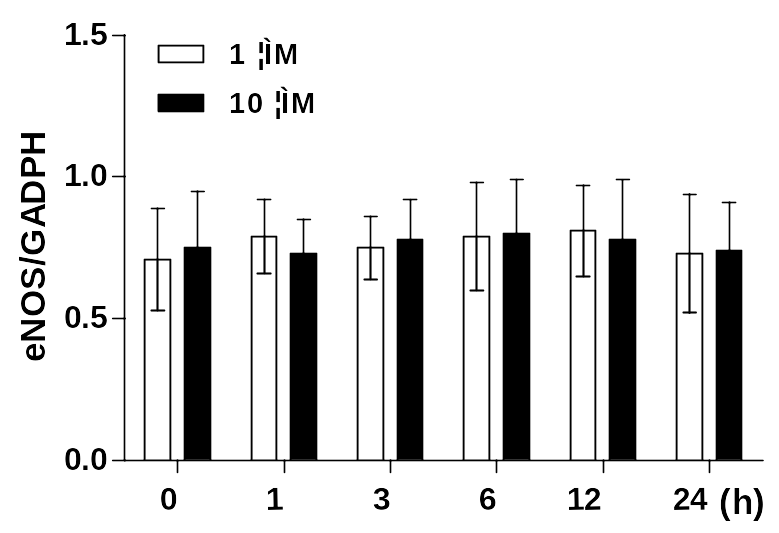

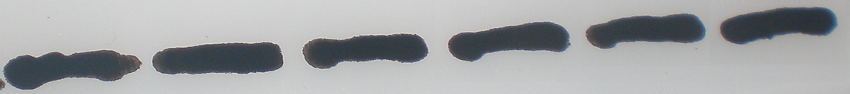

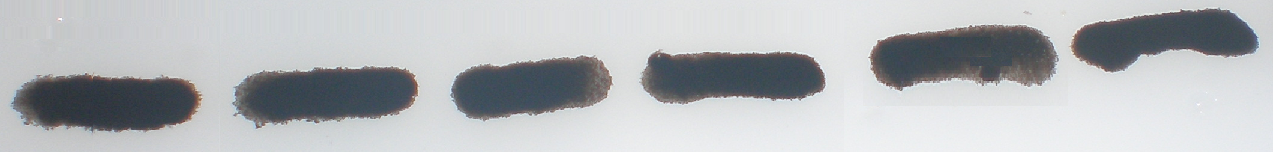

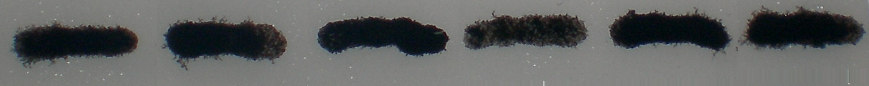


**Fig. S2** Effect of bilobalide on OEC eNOS expression. Rat BM OECs that had been incubated in serum-free EBM-2 for 12 h were exposed to bilobalide and collected at the indicated time points to test eNOS. Upper, representative Western blotting bands; bottom, statistics: *n*=3 per group.


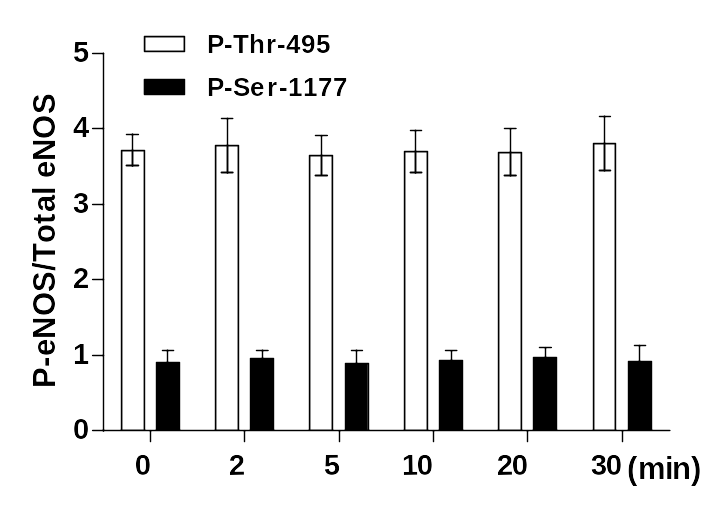


**0**

**2**

**5**

**30 (min)**

**Total eNOS**

**P- eNOS (Thr-495)**

**P- eNOS (Ser-1177)**

**GADPH**

**10**

**20**


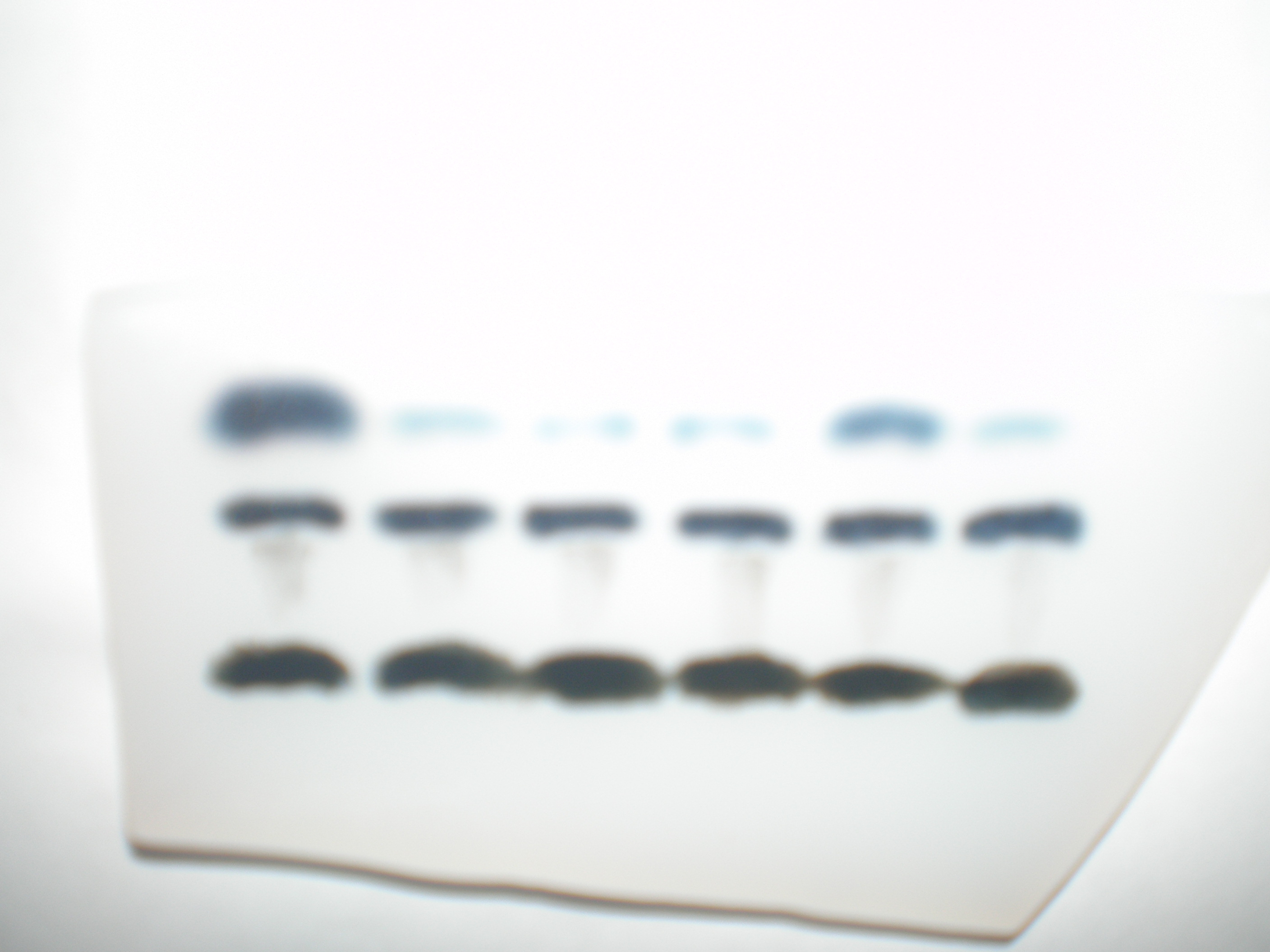

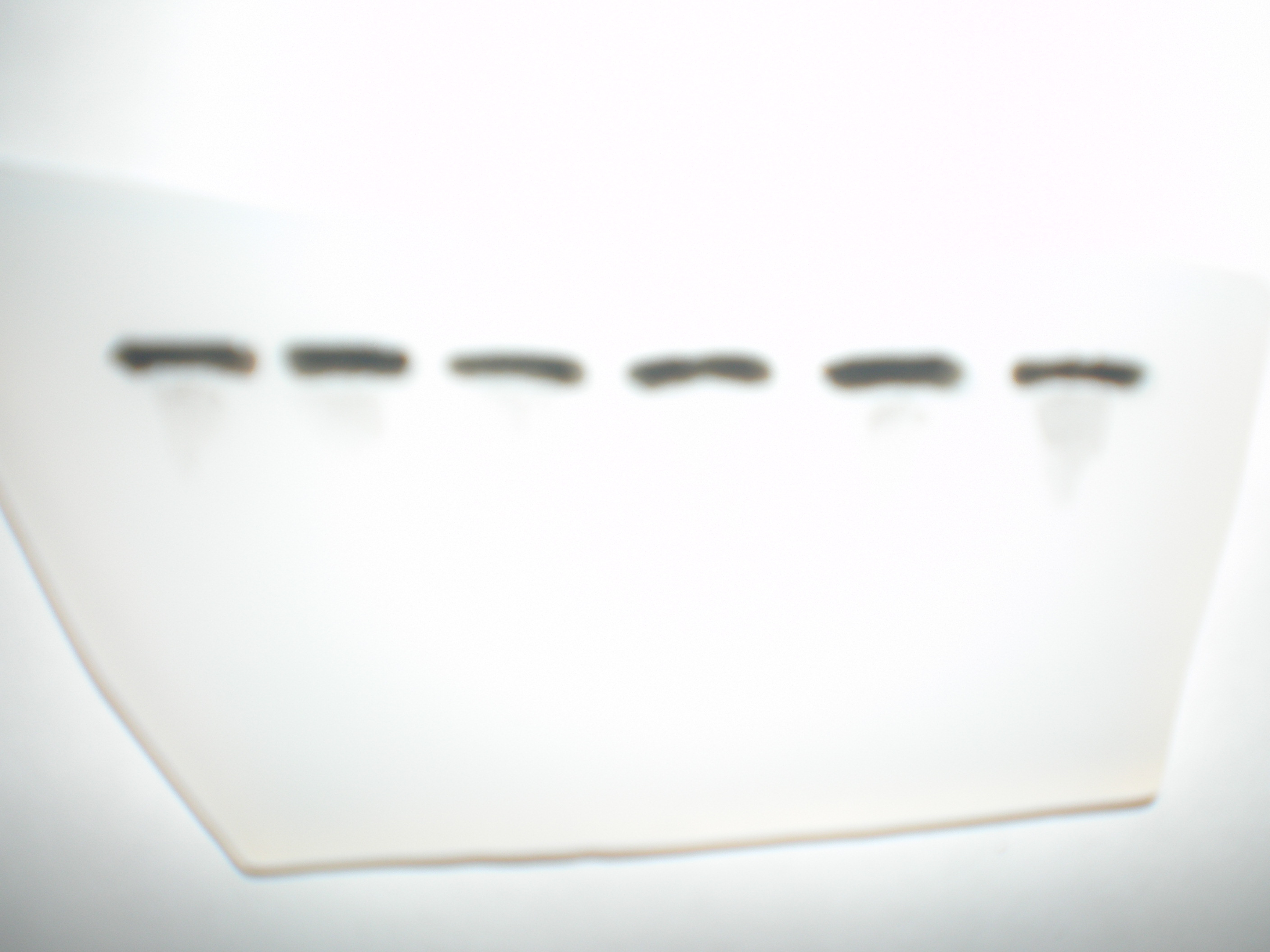

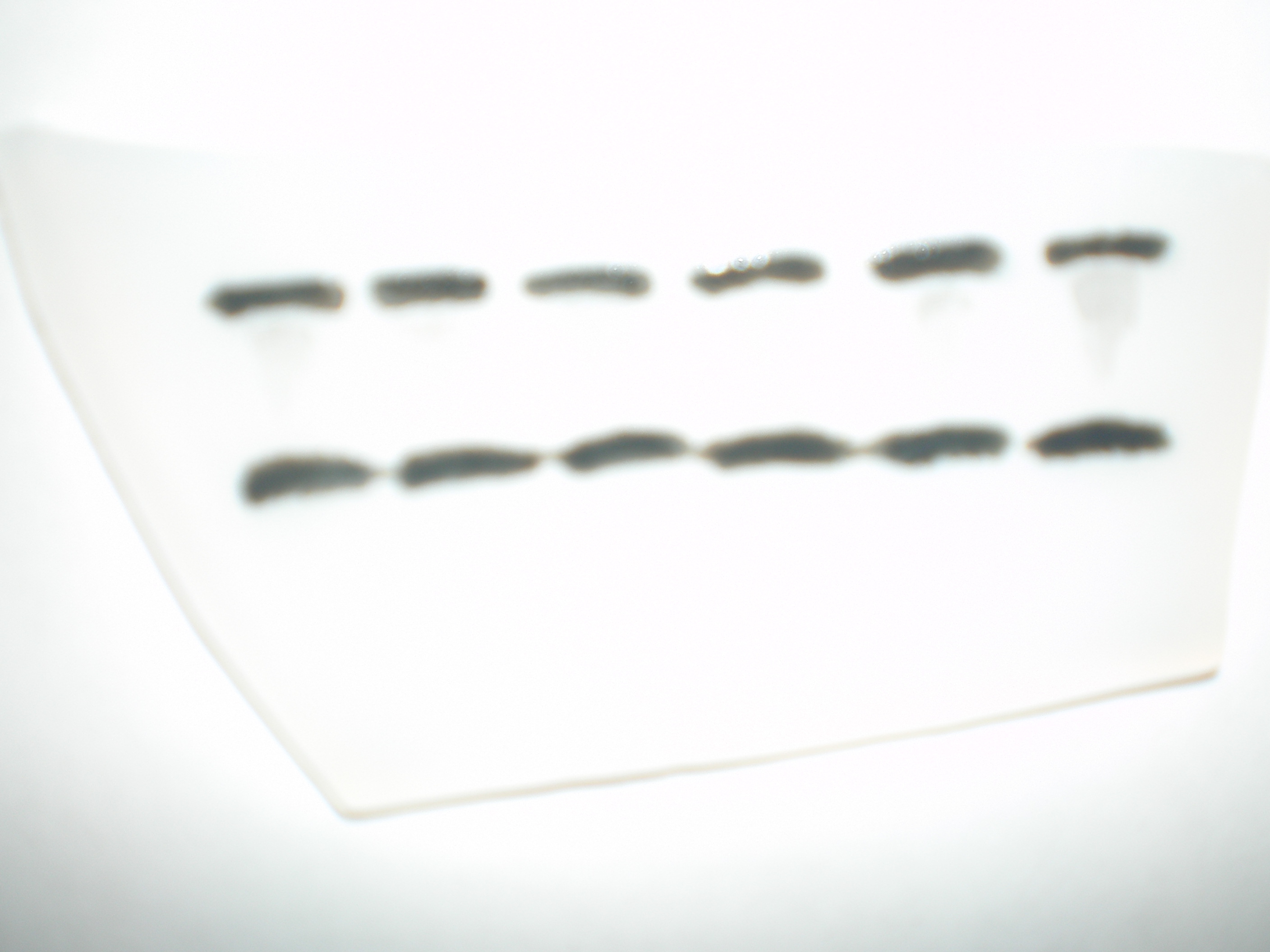

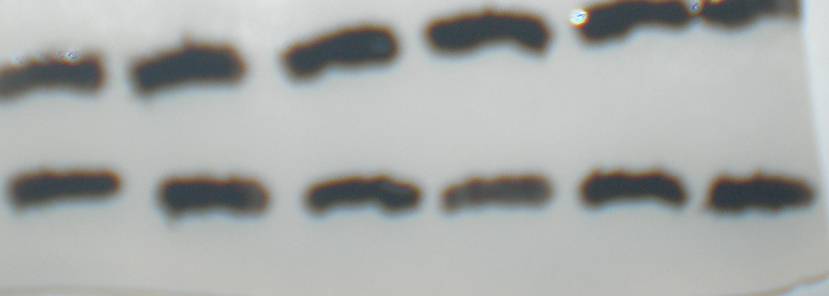


**Fig. S3** Quiescent OEC eNOS phosphorylation at Thr-495 and Ser-1177. Rat BM OECs incubated in serum-free EBM-2 were collected at the indicated time points to test phosphorylated eNOS (P-eNOS) at Thr-495 or Ser-1177. Upper, representative Western blotting bands; bottom, statistics: *n*=3 per group.
